# Emergency management with resection versus proximal stoma or stent treatment and planned resection in malignant left-sided colon obstruction

**DOI:** 10.1186/s12957-016-0994-2

**Published:** 2016-08-30

**Authors:** Emma Öistämö, Fredrik Hjern, Lennart Blomqvist, Ylva Falkén, Klas Pekkari, Mirna Abraham-Nordling

**Affiliations:** 1 Department of Clinical Sciences, Division of Surgery, Danderyd Hospital, Karolinska Institutet, Stockholm, Sweden; 2 Department of Diagnostic Radiology, Department of Molecular Medicine and Surgery Karolinska University Hospital Solna and Karolinska Institutet, Stockholm, Sweden; 3 Division of Coloproctology, Center of Digestive Diseases, Karolinska University Hospital, Stockholm, Sweden; 4 Department of Molecular Medicine and Surgery, Karolinska Institutet, Stockholm, Sweden

**Keywords:** Colon cancer, Emergency resection, Stoma, Self-expanded metallic stent (SEMS)

## Abstract

**Background:**

Emergency surgery for colon cancer, as a result of obstruction, has been vitiated by a high frequency of complications and poor survival. The concept of “bridge to surgery” includes either placement of self-expanding metallic stents (SEMS) or diverting stoma of an obstructing tumour and subsequent planned resection. The aim of this study was to compare acute resection with stoma or stent and later resection regarding surgical and oncological outcomes and total hospital stay.

**Methods:**

This is a retrospective cohort study. All 2424 patients diagnosed with colorectal cancer during 1997–2013 were reviewed. All whom underwent acute surgery with curative intention for left-sided malignant obstruction were included in the study.

**Results:**

One hundred patients fulfilled the inclusion criteria. Among them, 57 patients were treated with acute resection and 43 with planned resection after either acute diverting colostomy (*n* = 23) or stent placement (*n* = 20). The number of harvested lymph nodes in the resected specimen was higher in the planned resection group compared with acute resection group (21 vs. 8.7; *p* = 0.001). Fewer patients were treated with adjuvant chemotherapy in the acute resection group than in the stoma group (14 % (8/57 patients) vs. 43 %, (10/23 patients; *p* = 0.024)). Patients operated with acute resection had a higher 30-day mortality rate and were more frequently left with a permanent stoma.

**Conclusions:**

Decompression of emergency obstructive left colon cancer with stent or stoma and subsequent curative resection appears safer and results in a higher yield of lymph node harvest, and fewer patients are left with a permanent stoma.

## Background

Colorectal cancer (CRC) is a common cancer with a yearly incidence in Europe of approximately 400.000 new cases [1]. Acute obstruction of the bowel is the initial presentation in 7–29 % of patients with CRC and often leads to an emergency surgical intervention. Acute presentation is more common in advanced stage disease and occurs more frequently in elderly patients [1, 2]. Left-sided colon obstructions due to tumours are particularly challenging due to risk of perforation in a dilated colon proximal to the tumour. Emergency surgery due to malignant obstruction of the left colon has been associated with a high frequency of complications and poor survival [2–5].

Surgical treatment of malignant colonic obstruction remains controversial especially regarding left-sided colonic obstruction [6–8]. The term bridge to surgery is defined as immediate treatment of the obstruction followed by delayed oncological surgery.

Self-expanded metallic stent (SEMS) and stoma are two bridge-to-surgery options. The rationale is to stabilise the condition of the patient by managing the acute obstruction and to create an elective situation for the tumour resection. This avoids malnutrition and a dilated colon and allows dedicated colorectal surgeons to perform the surgery to improve outcome. Disadvantages with SEMS include perforation, re-obstruction, and stent migration [9, 10]. For stoma in the emergency setting, the disadvantages includes that 2/3 of patients are reported to be left with a permanent stoma and significantly higher morbidity rates compared to patients undergoing elective surgery for left-sided colon carcinoma [6, 11, 12].

The aim of this study was to perform an evaluation of short term outcomes of acute resection versus bridge to surgery with stoma or stent in acute left-sided colonic cancer obstruction with focus on post-operative morbidity, hospital stay, and oncological outcome.

## Methods

### Patients

The study is a retrospective single-centre cohort study including all patients who had surgery for colon cancer from January 1997 to December 2013 at Danderyd University Hospital, Stockholm, Sweden. Patients were retrieved from the Regional Colon Cancer Register and the National Colon Cancer Register, where data of all cancers are reported from both clinicians and pathologists. The register has previously been shown to have high validity and is continuously updated [13].

**Fig. 1:**
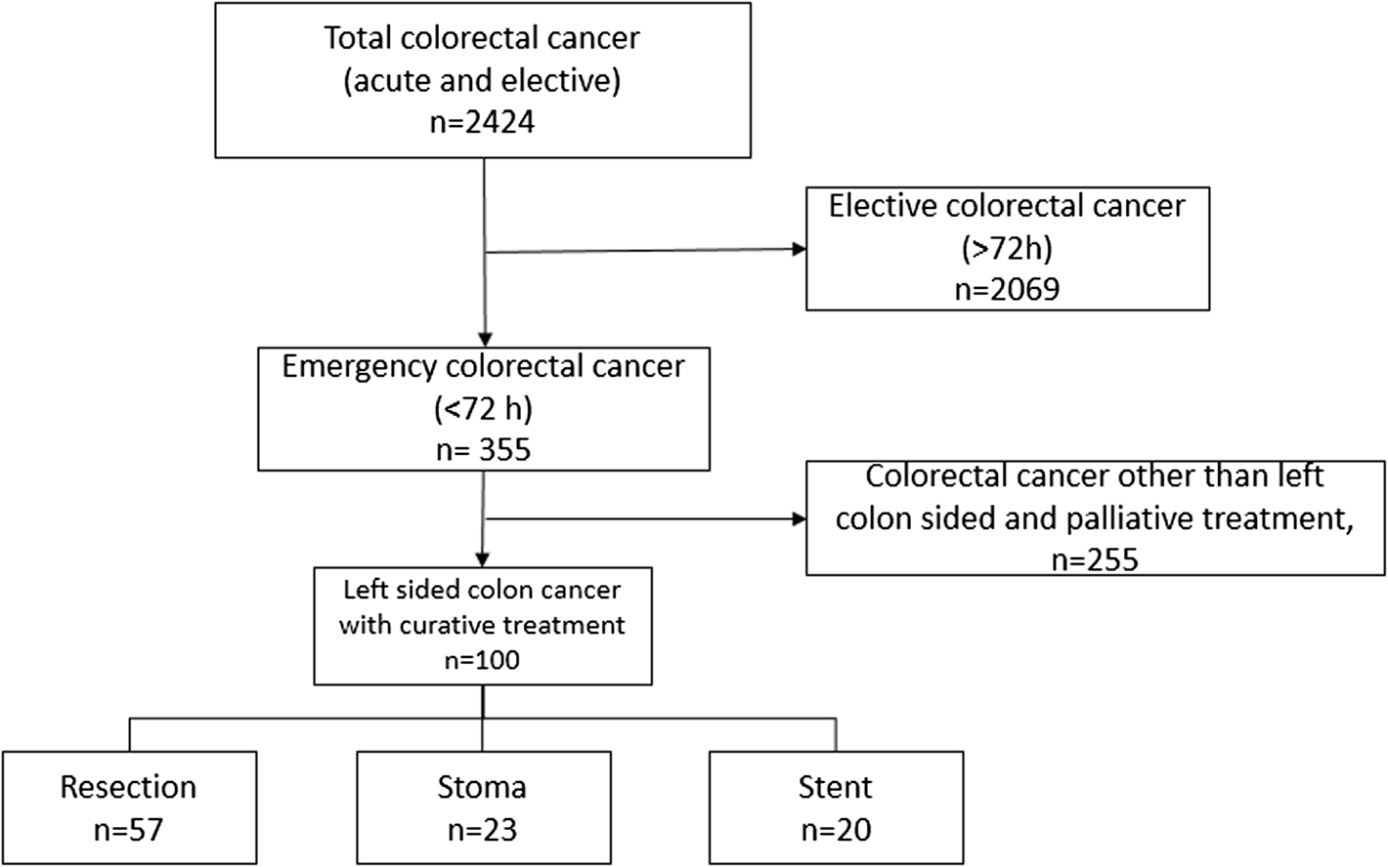
Study flow diagram

Patients with rectal cancer (<15 cm from the anal verge), right-sided tumours, elective procedures, acute surgery due to other reasons than obstruction, and palliative procedures and all who were not finally biopsied confirmed as colonic adenocarcinomas were excluded (Fig. 1). Acute surgery was defined as surgery within 72 h of the first admission with no previous knowledge of the disease.

The review of cancer registry data and patient files were reviewed by one surgeon (EÖ), who had not been involved in the treatment of the patients. Variables recorded included gender, age, co-morbidity, American Society of Anesthesiologists (ASA) classification, tumour location (left-sided if located between the splenic flexure and the rectosigmoid junction), stage of tumour, TNM-classification and metastatic disease (American Joint Committee on Cancer (AJCC)), treatment characteristics (type of surgery, oncological treatment), and complications.

Complications were defined as all complications added together at each hospital stay and 30 days after surgery until 30 days after the final tumour resection or stoma were reversed.

The total hospital stay was defined as all hospital days (all episodes of surgery) until 30 days after surgery where the tumour was resected or stoma were reversed, for instance, in the stoma group, episode of stoma surgery, resection surgery, and the episode when stoma was reversed.

The study was approved by the local ethics committee of the Karolinska Institutet (KI 2012/897-31/1).

### Statistics

The statistical data were analysed using Statistica, SPSS 22. Numerical data were compared by independent *t* test and nominal data by Fisher’s exact test or chi-squared test. When significant result was found comparing the three groups, a post hoc analysis was executed with Anova, Tukey HSD, Games-Howell, and Bonferroni.

## Results

In all, 2424 patients underwent colorectal cancer surgery during the study period. Of them, 2069 had elective or semi elective surgery, and 355 patients had emergent surgery (<72 h after admission to hospital). Patients not treated with curative intention or cancer in other locations than the left colon were excluded (*n* = 255) (Fig. 1). The final study group constituted of 100 patients. Patients having acute resection of the tumour were defined as the acute resection group and comprised of 57 patients. These patients had either a primary anastomosis and in some cases a protective stoma or a Hartmann’s procedure performed at time of primary surgery. The remaining 43 patients with planned resection consisted of two subgroups. First, in 23 patients, an emergency operation with a proximal stoma was performed and the tumour resection was done electively in a subsequent procedure (stoma group). Finally, 20 patients underwent treatment with SEMS and subsequent tumour resection (stent group, Fig. 1).

Patient characteristics are shown in Table 1. There were no significant difference between the three groups according to sex, co-morbidity (ASA classification), and time from onset of obstruction to surgery. The patients in the acute resection group where significantly older than the patients in the stoma group (74 vs. 67; *p* = 0.040).

**Table 1:** Baseline characteristics of 100 patients included in the study

	Acute	Staged resection		*p* value
	Resection	Stoma	Stent	
	*n* = 57 (%)	*n* = 23 (%)	*n* = 20 (%)	
Sex ratio (M/F)	31:26 (55/46)	13:10 (57/43)	7:13 (35/65)	0.274
Age (year)	74 ± 12	67 ± 12	71 ± 10	0.040
ASA				0.144
1	12 (21)	1 (4)	7 (35)	
2	18 (32)	13 (57)	6 (30)	
3	24 (42)	7 (30)	7 (35)	
4	3 (5)	2 (9)	0 (0)	
Days of obstruction	1.3	1.2	1.5	0.552

Values in parentheses are percentage unless otherwise indicated. Values are mean ± SD. The association with sex, the Pearson chi-square, and the association with the age and days of obstruction ANOVA was used. Association with ASA and the linear-by-linear association were used

**Fig. 2:**
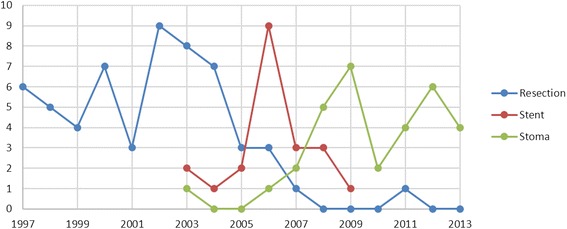
Numbers of the different procedures during each year during the study period 1997–2013

However, in the acute resection group, most of the patients underwent surgery during 1997–2007. Over the years 2003–2008, the SEMS procedure was performed and between the years 2007 and 2013, bridge to surgery with stoma and staged resection was performed (Fig. 2).

### Lymph nodes harvested

Lymph nodes harvested were significant less in the acute resection group compared to both the stoma and stent groups (mean 8.7 vs. 21 and 21; *p* = 0.001). The rate of patients with >12 lymph nodes harvested were 70 and 65 %, respectively, in the stoma and stent groups, compared to only 23 % in the acute resection group (*p* = 0.001).

### Adjuvant therapy

Less proportion of patients in the acute resection group than in the stoma group (8/57 vs. 10/23 patients; *p* = 0.024) received adjuvant chemotherapy. There was no significant difference between the acute resection group and the stent group (8 vs. 4 patients; *p* = 0.486; Table 2).

**Table 2:** Characteristics of resection, stoma, and stent groups

	Acute	Staged resection		*p* value
	Resection	Stoma	Stent	
	*n* = 57 (%)	*n* = 23 (%)	*n* = 20 (%)	
Tumour location				0.094
Splenic flexure colon	11 (19)	5 (22)	2 (10)	
Descending colon	7 (12)	7 (30)	2 (10)	
Sigmoid colon	39 (68)	10 (44)	15 (75)	
Rectosigmoid colon	0 (0)	1 (4)	1 (5)	
Number of operations	1.1 ± 0.4	2.5 ± 0.5	2.0 ± 0.2	<0.001^a^
Time between the first surgery and resection (days)		37.2 ± 25.8	40.7 ± 52.4	
Tumour stage				0.084
pT1	0 (0)	0 (0)	0 (0)	
pT2	4 (7)	0 (0)	1 (5)	
pT3	43 (75)	14 (61)	11 (55)	
pT4	10 (18)	9 (39)	8 (40)	
Lymph node(LN) mean number	8.7 ± 7.9	21 ± 17.4	21 ± 16.1	<0.001^b^
				<0.001^c^
LN ≤12	40 (70)	7 (30)	7 (35)	
LN >12	12 (23)	16 (70)	13 (65)	
Missing data	5 (7)	0 (0)	0 (0)	
Number of patients with metastatic LN (total: N1:N2)	25; 15:10	11; 4:7	11; 5:6	0.606
Missing data	0	2	0	
				0.773
R0	55 (96)	23 (100)	19 (95)	
R1	2 (4)	0 (0)	1 (5)	
Number of patients with adjuvant therapy				0.024^d^
Yes	8 (14)	10 (43)	4 (20)	
No	48 (84)	13 (57)	15 (75)	
Missing data	1 (2)	0 (0)	1 (5)	
Hospital stay (days) mean number				
Total	14.9 ± 8.1	24.1 ± 14.0	14.3 ± 6.8	0.001^e^
First operation	13.1 ± 7.6	11.5 ± 7.9	7.5 ± 4.7	0.014^f^
Surgeon level				0.001^g^
Surgical trainee	1 (2)	0 (0)	0 (0)	
Specialist in general surgery	29 (51)	0 (0)	1 (5)	
Specialisation in colorectal surgery	27 (47)	23 (100)	19 (95)	

Values in parentheses are percentage unless otherwise indicated. Values are mean ± SD

^a^Number of operation; a statistically significant difference observed between the resection group and stoma group *p* < 0.001, the resection group and stent group *p* < 0.001, and the stoma group and the stent group *p* = 0.003

^b^Lymph node mean; a statistically significant difference between resection group and stoma group *p* < 0.001 as well the stent group *p* < 0 .001; no significant differences between stoma group and stent group *p* = 1.000

^c^Lymph node ≤12 and >12; *p* < 0.01

^d^Adjuvant therapy; a significant difference between resection group and stoma group, *p* = 0.024. No statistically significant differences between the resection group and stent group *p* = 0.486

^e^Total hospital stay; a significant between the reference group stoma compared with resection group *p* = 0.018 and compared with stent group *p* = 0.015. Reference group resection compared with stent group *p* = 0.930

^f^Hospital stay at the first operation; a significant difference between the reference group resection and compared with stent group *p* = 0.010; comparing the resection group and the stoma group shows no statistical significant *p* = 0.658. No significant between the stoma group and stent group *p* = 0.164

^g^Surgeon level; a significance between the resection group and stoma group *p* = 0.001 as well as between the stent group *p* = 0.001. No significant between the stoma and stent group *p* = 0.465

### Surgeon level

In 95 % (19/20) of the subsequent resection procedures performed after an initial stent, the most experienced surgeon involved in the procedure had at least 2 years of colorectal specialisation, and in 100 % (23/23) after an initial stoma decompression, compared with only 47 % (27/57) of the acute resections (*p* < 0.001; Table 2).

### Number of operations

There were significantly fewer surgical procedures per patient in the acute resection group compared to the stoma group (1.1 vs. 2.5 operation; *p* < 0.001) and also compared to the stent group (1.1 vs. 2.0 operation; *p* < 0.001). A significance was also seen between the stoma and the stent groups (2.5 vs. 2.0; *p* = 0.003).

### Total hospital stay

Total hospital stay was longer for patients in the stoma group compared to patients treated with acute resection and stent (*p* = 0.001; Table 2).

### Characteristics and staging of tumours

Tumours penetrating the muscularis propria (T3 and T4) were the most common in all three treatment groups (Table 2). There were no significant differences between the groups.

### Surgical complications

Complications, i.e. Clavien-Dindo I–V [14], did not differ significantly between the three groups (20 vs. 8 vs. 6 patients; *p* = 0.207) (Table 3). However, in the acute resection group, 15 patients (26 %) had serious complications III–V; of them, seven patients (12 %) died within the first hospital stay, five due to circulatory failure, one out of sepsis, and one of unknown reason. No fatal outcomes were observed in patients that underwent acute stoma and stent and subsequent planned resection.

**Table 3:** Severity of surgical complication

	Acute	Staged resection		*p* value
	Resection	Stoma	Stent	
	*n* = 57 (%)	*n* = 23 (%)	*n* = 20(%)	
Number of complication	20 (35)	8 (35)^a^	6 (30)	0.207^b^
Clavien-Dindo grade				
I				
II	5	1	3	
IIIa + b	8	7	2	
IVa + b	0	0	1	
V	7	0	0	

^a^Three complications were related to the first operation, stoma creation

^b^Analysis of complications with Pearson chi-square test and linear-by-linear association. Values in parentheses are percentage unless otherwise indicated

#### Permanent stoma

The definition of permanent stoma is that the patient had an existing stoma at time of death or at the time when the study was completed. Overall, 25/57 (44 %) in the acute resection group received a stoma at the first séance of which 24 were operated with Hartmann’s procedure and received a colostomy. Five of them had a second procedure with stoma closure and anastomosis (mean 203 days after first surgery, data not shown in table). The last patient received an ileostomy that was never closed. Therefore, in the acute resection group, 20 (35 %) patients ended up with permanent stoma. Among patients having a planned resection, only 9/43 patients (21 %) were left with a permanent stoma. In the stoma group 22 (96 %), patients had their stoma reversed and 1 (4 %) patient were left with a permanent stoma. Among the stent group, 10 patients had a direct anastomosis after the tumour resection and never received a stoma after, and 8 patients (40 %) received stomas that were never reversed (data not shown in table).

## Discussion

Emergency surgery for obstructed left-sided colon cancer is associated with poor results [15, 16], and the method of creating a bridge to surgery from the emergency period to elective management remains controversial. In the present study staged resection, diversion with either stoma or SEMS and subsequent curative resection improved lymph node harvest. In the acute resection group, the insufficient visual field due to edema and bowel extension adds difficulties to resect enough specimens. The high number of lymph nodes harvested (>12 nodes harvested) in the bridge to surgery group mirrors how the surgical and oncological technique has evolved over time. In the beginning of the twenty-first century, the concept of complete mesocolic excision (CME) for colonic cancer was introduced [17]. The CME technique in colon cancer surgery aims at a specimen with intact layers and a maximum of lymph node harvest, and this technique is easier to implement on patients with elective condition. The bridge to surgery groups resemble elective patients and are thus eligible for the CME technique. This is further translated into lower local recurrence rates and better overall survival. Another factor is that almost all bridge to surgery patient’s resection surgery was performed by a surgeon with at least 2-year sub-specialisation in colorectal surgery.

Although, there were no significant difference between the three groups according to sex, co-morbidity, and time from onset to surgery. There is a remarkable high proportion of severe complications (Clavien-Dindo 3 or 4) (*n* = 15, 26 %) in the acute resection group, including a 12 % mortality rate compared to 0 % in the planned resection group; these results are comparable with other reports in the literature [18, 19]. The reason for this might be a coincidence, but taking into account the stage of cancer in this group and that the surgery was performed in the late 90s, when post-operative care was not as advanced as today, might explain this finding.

The rate of post-operative adjuvant therapy differed between the acute resection and the stoma group (*p* = 0.024), and overall, a trend was noted to give adjuvant treatment in the planned resection groups compared with the acute resection group (20–43 vs. 14 %). Surgery in the stoma and SEMS group was mainly performed during 2003–2012, and multi-disciplinary team conferences (MDT) were introduced in our hospital during this time period. During the late 90s, when most of the emergency resections were made, these patients were not frequently discussed at MDT conferences (Fig. 2). As the MDT conferences are a quality measure, most likely more patients in the resection group could have become candidates for adjuvant chemotherapy treatment [20]. Another reason might be the high complication rate among patients having an acute resection that is a contraindication to adjuvant chemotherapy treatment.

Earlier, there has been a trend towards one-stage surgery. Since introducing “bridge to surgery”, the debate on how to treat these patients has emerged. Although the optimal treatment for patients with left side obstruction in CRC remains controversial, most studies report better results for staged management [16, 21–23]. Kronborg et al [4] demonstrated in the only randomised trial of emergency colostomy versus acute resection in patients with left-sided colonic or rectal obstruction that the only advantage with acute resection was a shorter hospital stay. In the present study, the SEMS group had shorter hospital stay after the initial operation. It might be due to the effectiveness of the endoscopic procedure as well as avoiding emergency surgery with stoma or emergency resection (Table 2). Besides being useful in acute malignant colonic obstruction both as a bridge to surgery SEMS is also a potential permanent treatment in the palliative setting [24]. Despite that, as it is shown in this report, SEMS is used in a lesser extent in non-palliative patients due to the risk of perforation, re-obstruction and stent migration. It has previously been shown that a significant proportion, 2/3 of the patients, receiving a staged operation never undergo reversal of the stoma [6, 9, 10]. Many studies have demonstrated that living with a colostomy influences the overall quality of life negatively such as depressive feelings, constipation, travel difficulties, and worry about noises [25]. Nevertheless, in the present study, only 4 % of the patients in the stoma group were left with a permanent stoma.

The definition of emergency surgery varies, making comparison with other trials difficult. Some studies defined “emergency obstruction” with no time limit but as long as the diagnosis of intestinal obstruction occurs [26]. In the present study, emergency was defined as surgery within 72 h of the first admission with no previous knowledge of the disease. An advantage with the present study is the extensive review of all patients’ records by one independent single surgeon, not involved in the treatment of the patients. Further, only patients who had intended curative treatment were included.

One weakness of our study is that it is a retrospective observational study and the material covers a long period of time. During this time period, both surgical and oncological management have been changed. The concept of specimen oriented surgery has emerged, with more emphasis on resection margins, lymph node harvest, and the pathologist quality of the specimen as assessed by histopathological examination. Even though these factors can influence the outcome, we thought there was a need to evaluate these different treatment strategies described in this study.

## Conclusions

For treatment of left colon obstruction caused by colonic carcinoma, a staged surgical resection, diversion with either stoma or SEMS, and subsequent curative resection appear safe and improve lymph node harvest, and fewer patients are left with a permanent stoma.

## Abbreviations

SEMS: Self-expanding metallic stents
CRC: Colorectal cancer
CME: Complete mesocolic excision
MDT: Multi-disciplinary team
